# Age, Purpose, and Perception With Regard to the First Dental Visit of Children of Goa State in India

**DOI:** 10.7759/cureus.68338

**Published:** 2024-08-31

**Authors:** Olando K D'souza, Paul Chalakkal, Siya Dukle, Ridhima B Gaunkar, Vernon Pires, Ida de Noronha de Ataide

**Affiliations:** 1 Department of Pediatric and Preventive Dentistry, Goa Dental College and Hospital, Bambolim, IND; 2 Department of Public Health Dentistry, Goa Dental College and Hospital, Bambolim, IND; 3 Department of Conservative Dentistry and Endodontics, Goa Dental College and Hospital, Bambolim, IND

**Keywords:** parental perception, infant oral health, early dental visit, preventive dental care, first dental visit

## Abstract

Background and aim: Early first dental visits (FDVs) are crucial for educating and informing parents about their children’s oral health. The age for the FDV is dependent on several factors and data regarding the same is unavailable in Goa state. The aim of the study was to determine the age, purpose, and parental perception with regard to the FDV of children in Goa State.

Methods: A cross-sectional study was conducted in the Department of Pedodontics and Preventive Dentistry for children reporting for their FDV. Data for the study was recorded from validated and pilot-tested questionnaires. Statistical analysis was performed using the STATA (version 17; StataCorp LLC, College Station, TX) software. Categorical variables were summarized using frequencies and percentages. Continuous variables were summarized using means and standard deviations.

Results: A total of 544 children were included in the study. The mean age of the FDV was 7.15 ± 2.98 years. Out of the total, 257 (47.24%) children reported with pain. The absence of dental problems was the reason why 485 (89.01%) children and their parents had not visited earlier. Most parents perceived the FDV to be important but were unaware of the ideal age for the same.

Conclusions: Children in Goa state have delayed FDVs. Although parents recognized the importance of early dental visits and oral health, their knowledge regarding the recommended age was found to be deficient.

## Introduction

The first dental visit (FDV) is a significant milestone in a child’s life [1]. The American Academy of Pediatric Dentistry (AAPD) and American Academy of Pediatrics (AAP) currently recommend that all children should have their FDV at the time of eruption of the first tooth and no later than 12 months of age [2,3].

The principal objectives of advocating early visits are to avert early childhood caries (ECC) and to identify and halt the advancement of any developing carious lesions. Additional goals revolve around educating parents about the importance of fluoride, dental hygiene practices, teething management, preventing accidents that could harm their child's face or teeth, and the link between diet and oral health [4,5].

The age and reasons for the FDV are influenced by factors such as socioeconomic status, educational attainment, and prior dental experience of the parents [6]. Parents play a significant role in imparting oral health-related knowledge and attitude in their children [7].

Children whose parents possess a positive approach towards the treatment of dental disease have better oral health [8]. However, a lot of parents choose to overlook their children's dental health because of their belief that permanent teeth eventually replace primary teeth, and also because they are unaware of the causes and treatment of ECC [9].

Indian parents have been reported to be less positive about their attitude on preventive dental care in children [10]. Focusing on initiatives to enhance health education regarding early dental visits is a major societal concern [7]. The Indian Society of Pedodontics and Preventive Dentistry (ISPPD) has adopted the AAPD guidelines on FDV and has recommended FDV of children before their first birthday [1].

Despite the positive health benefits, the majority of the population is unaware of the importance of early preventive dental visits, and most children fail to have their FDV within one year of age [7,11]. In India, very few children visit a dentist before four or five years of age and the FDV is usually a troubled visit with the child reporting most often with pain or swelling. Consequently, pediatric dentists are unable to provide timely preventive guidance [1]. To organize public awareness campaigns, it is crucial to assess the age of children and the most frequent reasons for their FDV along with parental perception of the same. Hence, the present study was aimed at determining the age, purpose, and perception with regard to the FDV of children in the state of Goa in India.

## Materials and methods

The study was conducted in the Department of Pedodontics and Preventive Dentistry in a government dental college and hospital. A convenience sample of children up to 13 years of age, visiting the department between November 2023 and April 2024 for their FDV, was included. The children had to be accompanied by their parents (or guardians in cases of deceased parents) who were willing to provide written informed consent to participate in the study. The study protocol was analysed and approved by the Institutional Review Board.

The age, purpose, and parental perception were recorded on a customized, researcher-administered, printed questionnaire, designed by the primary investigator. The questionnaire underwent face and content validation by 10 independent subject experts. A cover letter describing the purpose of the study and the validation process along with face and content validation forms were provided to each validator personally. Doubts pertaining to the same were cleared either personally or via telephonic communication. The duly filled validation forms were collected from the validator within 24-48 hours.

For face validation, each validator had to read the questionnaire in detail and mention either ‘Yes/No’ for each question depending on whether they agreed with the criteria for the objective of the construct or not. The validator could also give recommendations and suggestions to improve the structure of the question(s) or questionnaire. The percentage agreement for each question was calculated using the following equation [12]:

$\frac{\text{Number of agreed raters per question}}{\text{Total number of raters per question}} \times 100$

Since all the questions had a percentage agreement between 90% and 100%, no modifications to the questions were performed at the end of face validation.

After completing the process of face validation, each validator filled out the content validation form. Each question had to be scored as either Essential, Useful but not essential, or Not necessary. The content validity ratio (CVR) of each question was then calculated using the following equation [13]:

$\mathbf{CVR}=\left({ne}-{N}/\mathbf{2}\right)/\left({N}/\mathbf{2}\right)$

where ne is the number of panelists indicating ‘essential’ (E) and N is the total number of panelists.

Since there were 10 experts for content validation, an item was considered to have content validity only if it had a CVR of above 0.62 [13]. Modifications suggested by experts were considered only for questions with a CVR <0.62. However, since the CVR score for all questions was greater than 0.62, no modifications to the questions were carried out.

The questionnaire subsequently underwent pilot testing among 40 parents to assess the time, comprehensiveness, appropriateness, and understandability of the questionnaire.

The questionnaire consisted of two parts. The first part recorded the sociodemographic characteristics of the participants and the second part recorded the purpose and parental perception of the FDV.

Statistical analysis was performed using the STATA (Statistical Software for Data Science by StataCorp LLC, College Station, TX; version 17) software. Descriptive statistics were conducted to understand the sample distribution. Categorical variables were summarized as frequencies and percentages. Continuous variables were summarized as means and standard deviations. The chi‑square test of independence was used to determine the significance of the results. The significance level was set at P <0.05.

## Results

Table 1 represents the sociodemographic data. A total of 544 children accompanied by their parents reported for their FDV. The mean age at which the children had their FDV was 7.15 ± 2.98 years. Out of the total, 301 (55.33%) first-born children and 400 (73.53%) mother-child dyads had reported for their FDV. The FDV was recommended by the mothers of 401 (73.31%) children. Regarding educational qualification, 263 (48.35%) parents who recommended the FDV had high school degrees. A total of 381 (70.04%) children resided in urban areas.

**Table 1:** Descriptive statistics of socio-demographic data

Demographic variable	Group	Frequency (n)	Percentage	Chi^2^	P-value
Age group	0-1 years	1	0.18	610.16	<0.001
	1-3 years	20	3.68		
	3-6 years	162	29.78		
	6-13 years	361	66.36		
		Mean	SD		
		7.15	2.89		
Gender	Male	283	52.02	0.889	0.346
	Female	261	47.98		
	Others	0	0		
Birth order of the child	First	301	55.33	674.24	<0.001
	Second	202	37.13		
	Third	33	6.07		
	Fourth	7	1.29		
	Fifth	1	0.18		
Accompanying person	Mother	400	73.53	120.47	<0.001
	Father	144	26.47		
	Guardian	0	0		
Dental visit was recommended by	Mother	401	73.31	122.36	<0.001
	Father	143	26.29		
	Guardian	0	0		
	Others	0	0		
Educational qualification of the person who recommended the dental visit	Non-formal education	19	3.49	535.21	<0.001
	Primary school degree	43	7.9		
	High school degree	263	48.35		
	Pre-university degree	153	28.13		
	Undergraduate degree	54	9.93		
	Postgraduate degree or higher	12	2.21		
	Unaware	0	0		
Place of residence	Rural	163	29.96	87.36	<0.001
	Urban	381	70.04		
	Total	544	100.0		

Table 2 represents the purpose and parental perception of the FDV. A total of 257 (47.24%) children reported pain as the reason for their FDV, and the main reason for late dental visits, as reported by 485 (89.01%) parents, was the absence of dental problems. The awareness regarding the importance of early dental visits was found in 380 (69.85%) parents. The number of parents who knew the age of eruption of the first primary tooth was 427 (78.64%). The importance of primary teeth and oral health was known to 493 (90.63%) and 502 (92.28%) parents, respectively. Successor teeth being unaffected following treatment in primary teeth treatment was known to 491 (90.26%) parents. The need for the FDV to be carried out following the eruption of all primary teeth was known to 356 (65.44%) parents. The need for treating primary teeth was expressed by 485 (89.15%) parents. Completion of dental treatment, regular follow-ups, and the willingness to visit the government hospital due to the availability of paediatric dentists was wanted by 504 (92.65%), 503 (92.46%), and 356 (65.44%) parents, respectively.

**Table 2:** Descriptive statistics of the purpose and parental perception of their child’s first dental visit

Question	Response	Frequency (n)	Percentage	Chi^2^	P-value
What is the purpose of your child’s first dental visit?	Routine dental visit/check-up	16	2.94	1659.61	<0.001
	Decayed teeth	109	20.04		
	Pain	257	47.24		
	Sensitivity	1	0.18		
	Crooked teeth	50	9.19		
	Missing teeth	5	0.92		
	Extra teeth/over-retained teeth	15	2.76		
	Swelling	67	12.32		
	Bleeding gums	4	0.74		
	Deposits on teeth	2	0.37		
	Halitosis	0	0		
	Injuries to teeth and supporting structures	13	2.39		
	Oral habits	1	0.18		
	Pus discharge from gums	1	0.18		
	Referral from a physician/paediatrician	0	0		
	Referral from a dental practitioner	0	0		
	Referral from school oral health camps	0	0		
	Others	3	0.55		
What is the reason for not bringing your child earlier for a dental check-up?	No time, working parents	15	2.79	3731.21	<0.001
	Lengthy waiting time, long appointments	1	0.19		
	Child would have to miss school	7	1.3		
	No dental office nearby	5	0.93		
	Dental treatment is expensive	0	0		
	Child is too young to cooperate	7	1.3		
	No dental problems so far	485	89.01		
	Fear of dental procedures	2	0.37		
	Adult teeth have not yet erupted	1	0.19		
	Milk teeth will fall off eventually	19	3.54		
	Unaware of available dental healthcare facilities for children	2	0.37		
Would an earlier visit to the dentist prevent the occurrence of dental disease?	Yes	380	69.85	383.66	<0.001
	No	154	28.31		
	Do not know	10	1.84		
Do you remember at what age did your child’s first milk tooth erupt?	Yes	427	78.64	178.12	<0.001
	No	116	21.36		
If yes, at approximately what age did the first tooth erupt?	0-6 months	11	2.58	573.66	<0.001
	6 months-1 year	357	87.82		
	More than 1 year	41	9.6		
What according to you is the correct age for your child’s first dental visit?	As soon as your child’s first tooth erupts	2	0.37	818.72	<0.001
	First birthday of your child	36	6.62		
	As soon as all milk teeth erupt	356	65.44		
	After all adult teeth erupt	8	1.47		
	If your child experiences a dental problem	142	26.1		
Are milk teeth important?	Yes	493	90.63	804.14	<0.001
	No	33	6.07		
	Do not know	18	3.31		
Does oral health influence your child’s general body health?	Yes	502	92.28	850.69	<0.001
	No	24	4.41		
	Do not know	18	3.31		
Do you think milk teeth need dental treatment?	Yes	485	89.15	763.59	<0.001
	No	38	6.99		
	Do not know	21	3.86		
Do you feel treatment of milk teeth will cause damage to permanent teeth?	Yes	11	2.02	795.89	<0.001
	No	491	90.26		
	Do not know	42	7.72		
What do you expect from the dentist?	Address only the problem for which you have got your child to the dentist	28	5.15	1330.29	<0.001
	Treat only teeth with major problems	10	1.84		
	Complete all the necessary dental treatments	504	92.65		
	Others	2	0.37		
Will you bring your child for check-up visits once the dental problem for which you visited the dentist is addressed?	Yes	503	92.46	1323.13	<0.001
	Only if the dentist asks for a check-up visit	4	0.74		
	Only if my child has a dental problem	8	1.47		
	No	29	5.33		
	Do not know	0	0		
Why did you choose to bring your child to the government hospital?	Financial reasons	17	3.13	788.70	<0.001
	Proximity of residence	6	1.1		
	Availability of specialized paediatric dentists	356	65.44		
	Recommended by others	129	23.71		
	Previous personal experience	36	6.62		
	Others	0	0		

## Discussion

Early dental visits serve as a foundation upon which oral health education and preventive measures can be built [14]. Despite the recommendations by professional organizations, very few children in India visit a dentist at the recommended age [1]. This study was conducted in the one and only dental college and hospital in Goa State which has a department catering to pedodontics and preventive dentistry. A validated questionnaire was utilized in this study (Table 3 and Table 4 in the Appendix).

The mean age of children at their FDV was found to be 7.15 ± 2.89 years. A majority of the children who had reported were in the age group of 6-13 years. Studies conducted in Lagos (Nigeria) and Greater Noida (India) showed similar results [15,16]. However, a study from Southern Poland reported a lower age for FDV (3.79 ± 1.82 years) [17]. In this study, only one child had reported for her FDV between six and 12 months of age, which is considered ideal [2,3]. Nearly similar results were obtained in Lagos (Nigeria) and Southern Poland [15,17]. These findings reflect the lack of awareness among parents about having to take their infants to the dentist at the recommended age as per the AAPD.

Literature indicates that the birth order of a child is a predisposing factor in influencing the willingness of parents to bring their children for their FDV [11]. In this study, the number of children who reported for their FDV decreased with an increase in the birth order, with a majority of the children being first-borns. This could have occurred because parents become meticulous with maintaining their children’s oral hygiene after unpleasant experiences with their first child, thus reducing the number of dental visits. Yun et al. reported a large percentage of mothers in China willing to attend the FDV of their first child, which the authors attributed to the attention first-born children received in Chinese families [11].

Most of the children in this study were accompanied by their mothers. Similar results were obtained in Bangkok (Thailand) and Jeddah (Saudi Arabia) [18,19]. In this study, most of the children had their FDV recommended by their mothers. These results imply that children are usually accompanied by the parent who recommended their FDV.

The educational qualification of a parent is significantly related to the child’s utilization of oral health services [20]. This study showed that nearly half of the parents had a high school degree. However, studies from Izmir (Turkey) and Chengdu (China) reported that higher levels of maternal education were associated with an increased likelihood of earlier dental visits [21,22].

It has been reported that the unequal distribution of health services resulted in lesser utilization of dental services in rural areas [20]. In this study, most of the children resided in urban areas. These findings indicate limited knowledge of parents residing in rural areas regarding the importance of oral health of children and the availability of paediatric dental facilities. Similar results were reported in Southern Poland [17].

It was found that pain was the predominant reason for the FDV, followed by dental caries. Similar findings were found in studies from Riyadh (Saudi Arabia), Lagos (Nigeria), and Chennai (India) [6,15,23]. However, these findings differed from those obtained from Jeddah (Saudi Arabia) and Wroclaw (Poland), where dental caries and routine dental checkups were reported as the most common reasons, respectively [19,24].

Physicians may be the first to observe oral conditions since they are usually the first to be approached by parents for their children [22]. In this study, none of the children had been referred by a physician or a paediatrician for their dental visit. This indicates that medical doctors are either unaware of the recommendations laid down by the AAPD or are untrained to address oral health conditions. A similar finding was reported in Izmir (Turkey) [21]. However, a large number of referrals from medical doctors were reported in Namakkal district (India) [7].

The primary reason for a delay in the child’s FDV was found to be a parental misconception that their children had no dental problems. A similar finding was reported in Lagos (Nigeria) and Jeddah (Saudi Arabia) [15,19]. A majority of the parents felt that an earlier visit to the dentist would have prevented the occurrence of dental diseases. It was also found that most parents felt that their child’s FDV should have occurred as soon as all the primary teeth had erupted. Lack of parental knowledge about the ideal age for the FDV was also reported in Namakkal district (India) and Malaysia [7,25].

In this study, the majority of the parents were aware of the age of eruption of their child’s first primary tooth. Out of these, most parents stated that their child’s first primary tooth erupted between six months and one year of age.

The knowledge parents impart to their children regarding oral hygiene has a long-term influence on the child’s oral health [26]. In this study, a majority of parents believed that primary teeth were important and that oral health had an influence on general body health. Similar results were obtained in a study conducted in Jeddah (Saudi Arabia) [19].

Untreated carious primary teeth may result in pain, infection, masticatory problems, tooth loss, loss of sleep, malnutrition, and growth retardation [27]. The results of this study revealed that most parents felt that primary teeth required dental treatment. This finding was inconsistent with that found in Puducherry (India), where nearly equal proportions of parents were in support and against primary tooth treatment [28]. It was also found that a majority of the parents believed that primary teeth treatment would not damage permanent successors, similar to the finding reported in Puducherry (India) [28].

Tickle et al. found that the majority of parents in the United Kingdom preferred to leave treatment decisions to the dentist [29]. The present study and a study from Malaysia reported similar findings [25].

Goa State has only one dental institute with a department exclusively dedicated to pediatric dentistry. This was probably the reason why most of the parents reported to the dental institute for their child’s FDV. Moreover, paediatric dentists are experts in creating a friendly, kind, and safe environment for children [30].

The study had two limitations. It did not assess the socioeconomic status of the child’s family. Moreover, the knowledge and attitude of parents towards dental procedures were not assessed. These factors could have influenced the age at which the FDV was undertaken. Future studies may be carried out to assess the knowledge regarding the age of the child’s FDV, the importance of primary teeth, and infant oral health, among medical students, obstetricians, gynaecologists, paediatricians, physicians, and nurses.

## Conclusions

Insufficient knowledge and delay in the FDV (mean age: 7.15 ± 2.89 years) were observed for children in Goa state, India. Although parents recognized the importance of early dental visits and oral health, parental knowledge about the age of the FDV, recommended by professional organizations, was lacking. Dental education programs for parents, the public, and society in general, on a regular basis, are much needed in Goa to increase their awareness about the importance of early dental visits and the recommended age. Moreover, a collaboration between paediatricians, gynaecologists, general practitioners, and dentists in Goa is desired in order to monitor the child’s oral health right from the time of their FDV.

## Appendices

**Table 3:** Socio-demographic details of the participants

Demographic variable	Group
Age group	0-1 years
	1-3 years
	3-6 years
	6-13 years
Gender	Male
	Female
	Others
Birth order of the child	First
	Second
	Third
	Fourth
	Fifth
Accompanying person	Mother
	Father
	Guardian
Dental visit was recommended by	Mother
	Father
	Guardian
	Others
Educational qualification of person who recommended the dental visit	Non-formal education
	Primary school degree
	High school degree
	Pre-university degree
	Undergraduate degree
	Postgraduate degree or higher
	Unaware
Place of residence	Rural
	Urban

**Table 4:** Purpose and parental perception on the FDV of their children FDV, first dental visit.

Question	Response
What is the purpose of your child’s first dental visit?	Routine dental visit/check-up
	Decayed teeth
	Pain
	Sensitivity
	Crooked teeth
	Missing teeth
	Extra teeth/over-retained teeth
	Swelling
	Bleeding gums
	Deposits on teeth
	Halitosis
	Injuries to teeth and supporting structures
	Oral habits
	Pus discharge from gums
	Referral from physician/paediatrician
	Referral from dental practitioner
	Referral from school oral health camps
	Others
What is the reason for not bringing your child earlier for a dental check-up?	No time, working parents
	Lengthy waiting time, long appointments
	Child would have to miss school
	No dental office nearby
	Dental treatment is expensive
	Child is too young to cooperate
	No dental problems so far
	Fear of dental procedures
	Adult teeth have not yet erupted
	Milk teeth will fall off eventually
	Unaware of available dental healthcare facility for children
Would an earlier visit to the dentist prevent the occurrence of dental disease?	Yes
	No
	Do not know
Do you remember at what age did your child’s first milk tooth erupt?	Yes
	No
If yes, at approximately what age did the first tooth erupt?	0-6 months
	6 months-1 year
	More than 1 year
What according to you is the correct age for your child’s FDV?	As soon as your child’s first tooth erupts
	First birthday of your child
	As soon as all milk teeth erupt
	After all adult teeth erupt
	If your child experiences a dental problem
Are milk teeth important?	Yes
	No
	Do not know
Does oral health influence your child’s general body health?	Yes
	No
	Do not know
Do you think milk teeth need dental treatment?	Yes
	No
	Do not know
Do you feel treatment of milk teeth will cause damage of permanent teeth?	Yes
	No
	Do not know
What do you expect from the dentist?	Address only the problem for which you have got your child to the dentist
	Treat only teeth with major problems
	Complete all the necessary dental treatments
	Others
Will you bring your child for check-up visits once the dental problem for which you visited the dentist is addressed?	Yes
	Only if the dentist asks for a check-up visit
	Only if my child has a dental problem
	No
	Do not know
Why did you choose to bring your child to the government hospital?	Financial reasons
	Proximity of residence
	Availability of specialized pediatric dentists
	Recommended by others
	Previous personal experience
	Others
